# Chinese version of the employee digital disconnection scale: development and validation

**DOI:** 10.3389/fpubh.2026.1762986

**Published:** 2026-02-03

**Authors:** Chunhua Jin, Yifei Li, Qi Wang, Na Zhang

**Affiliations:** School of Business, Beijing Information Science and Technology University, Beijing, China

**Keywords:** Chinese organizational context, digital disconnection, information and communication technologies (ICTs), measurement, mental health, scale

## Abstract

**Introduction:**

In the digital workplace, “always on” is progressively increasing the psychological burden on employees and having a negative impact on their mental health. As a proactive self-regulation strategy, digital disconnection aims to alleviate the psychological stress caused by continuous connection by intentionally limiting their use of electronic devices and digital applications. Existing research has preliminarily defined the concept of digital disconnection, but discussions on this topic remain insufficient, particularly given the lack of effective measurement tools applicable to the Chinese organizational context. This has led to inadequate exploration of the mechanisms through which digital disconnection operates among employees in China’s collectivist culture.

**Methods:**

Therefore, this study investigates the structural dimensions and measurement of employee digital disconnection in the Chinese organizational context through the questionnaire survey method.

**Results:**

By analyzing 795 questionnaires in two stages, it was found that the Chinese Version of the Employee Digital Disconnection Scale (Chinese-EDDS) includes four dimensions: digital disconnection from private information and communication technologies (ICTs) outside work (DD POW), digital disconnection from work-related ICTs outside work (DD WOW), digital disconnection from private ICTs during work (DD PDW), and digital disconnection from work-related ICTs during work (DD WDW), demonstrating good reliability and validity.

**Discussion:**

This study provides a reliable quantitative tool for researching employee digital disconnection in the Chinese organizational context, thereby offering a basis for organizations to develop supportive tools and training programs to improve the mental health of employees.

## 1 Introduction

Information and communication technologies (ICTs) have deeply penetrated the daily work of employees, demonstrating significant advantages in improving communication and flexibility, but they have also introduced new challenges, such as mental health risks derived from continuous connection. The widespread adoption of instant communication tools such as WeChat Work and DingTalk has led to work-related information intruding into private domains, resulting in phenomena such as being online over time after work hours. Similarly, excessive digital connectivity during work hours negatively affects employees, as the constant influx of emails, phone calls, or instant messages may disrupt their workflow (1), force them to multitask (2), and lead to a sense of technological overload (3, 4). This may bring the stress of being on call at all times to employees, thereby having detrimental effects on employees’ mental health, performance, commitment, and job satisfaction (4, 5). In order to alleviate the negative impact of continuous connection on employees’ mental health, rebuild the boundary between work and life, and restore psychological resources, digital disconnection has emerged as a strategy in which individuals intentionally limit their use of electronic devices and digital applications (6). As more researchers focus on the formation mechanisms and impacts of digital disconnection, the need to quantify this behavior effectively has become increasingly urgent.

Previous research has preliminarily defined the concept of digital disconnection, but some shortcomings still exist. First, previous studies on digital disconnection have been mostly qualitative, employing methods such as crawlers (7), participatory or nonparticipatory observation (8), ethnography (9), and interviews to explore the definition, motivations, and strategies of digital disconnection. However, quantitative analysis and empirical research to verify the relationships between digital disconnection and other variables such as mental health, are lacking (10). Second, previous study has focused on the digital disconnection of employees (10), but their samples mainly came from the European public sector, lacking cross-cultural tests and diverse samples. Our research was conducted in the Chinese context, without industry restrictions, and the samples were more diverse. Third, current studies on digital disconnection predominantly originate from Western cultural contexts that emphasize individualism. However, China’s profound collectivist and high-context communication traditions may fundamentally reshape employees’ cognitive organization of disconnection strategies. The Western conceptual distinction between “technological restrictions” and “communication” behaviors may not hold, or may manifest differently in the high-context Chinese culture, as Chinese behaviors are closely related to specific environments. Employees might experience stronger guilt and anxiety about disconnection because of fears of being detached from the collective or appearing unsociable. Given the significant cultural differences between China and the West, the lack of localized research in China has left the unique forms and roles of digital disconnection in Chinese organizations systematically unrecognized and unrefined, thereby weakening the contextual adaptability of digital disconnection theory construction and its explanatory power for Chinese management practices.

Our research is not merely about directly translating the Employee Digital Disconnection Scale (EDDS) in the context of Chinese culture. Its core theoretical contribution lies in the crucial theoretical expansion and structural improvement of the construct of employees’ digital disconnection based on Chinese collectivist culture. Specifically, our research reintroduced an additional dimension (digital disconnection from private ICTs outside work, DD POW), which was excluded in the original European study. It reveals that in China’s relations-intensive society, employees must also proactively manage the “erosion of work recovery resources by private social networks.” Then, this study tested the Chinese Version of the Employee Digital Disconnection Scale’s reliability and validity among a large sample of Chinese employees, aiming to provide an effective measurement tool for further exploring how employee digital disconnection affects the mental health of Chinese employees in the future. It also provides scientific support for organizations to design management strategies with psychological protective effects.

## 2 Literature review

### 2.1 Digital disconnection

Nassen et al. (11) proposed a definition of digital disconnection through a systematic review: “Digital disconnection is a deliberate (i.e., chosen by the individual) form of non-use of devices, platforms, features, interactions, and/or messages that occurs with higher or lower frequencies, and for shorter or longer periods of time, after the initial adoption of these technologies, and with the aim of restoring or improving one’s perceived overuse, social interactions, psychological well-being, productivity, privacy and/or perceived usefulness”. This definition not only encompasses the specific behavioral strategies individuals adopt but also deeply reveals the underlying motivations, providing a comprehensive perspective that lays a solid foundation for subsequent research.

To date, most research on digital disconnection has examined only one behavior at a time, primarily by evaluating the effects of interventions, such as using digital detox apps (12), blocking online distractions (13), or batching smartphone notifications (14). While these studies contribute to understanding the implications of partial disconnection, employees can engage with multiple ICTs disconnections in various ways, making it imperative to adopt a more comprehensive approach to measuring disconnection behaviors. Therefore, Verlinden et al. (10) developed the EDDS. This scale incorporates employees into the study of digital disconnection, measuring their intentional disconnection behaviors rather than the degree of disconnection. By combining the content of disconnected ICTs (work-related and private) with the scenarios of disconnection behaviors (during work and outside work), they identified four contexts of employee digital disconnection: digital disconnection from private ICTs outside work (DD POW), digital disconnection from work-related ICTs outside work (DD WOW), digital disconnection from private ICTs during work (DD PDW), and digital disconnection from work-related ICTs during work (DD WDW). Verlinden et al. (10) argued that the first context (DD POW) is not unique to employees but applies to any media user, thus focusing on the latter three contexts and developing the EDDS for these three scenarios. However, the context of DD POW should also be included in research. Although Verlinden et al. (10) did not address it on the grounds that this context “is not specific to employees,” this precisely indicates that employees commonly face such disconnections. Therefore, to understand employees’ digital disconnection behaviors more comprehensively, it is crucial to develop measurement tools that encompass this dimension.

The EDDS developed by Verlinden et al. (10) captures employees’ engagement in digital disconnection, advancing the field. However, this scale has several limitations. The participants were mainly from the public sector, which restricts the generalizability of the findings. Additionally, the participants were from Europe, where Western countries exhibit individualistic tendencies, whereas the collectivist culture in China emphasizes that individual interests are subordinate to collective interests. People will experience stronger social expectations (15), so it may be difficult for employees to achieve digital disconnection. Existing studies have shown that national culture can significantly influence employees’ behavioral choices (16). Therefore, exploring the manifestations of employees’ digital disconnection behavior in different cultural contexts is crucial for forming context-specific management approaches.

Scholarly research on digital disconnection has been conducted within the Chinese context (17), indicating that digital disconnection is a significant phenomenon of interest in China. Therefore, this study was conducted among Chinese employees without restricting participants’ industries to increase sample diversity.

### 2.2 Distinction and connection with similar constructs

Digital disconnection has certain distinctions and connections with technology non-use, media resistance, digital detox, and digital minimalism.

#### 2.2.1 Technology non-use and media resistance

Digital disconnection evolved from the concepts of technology non-use and media resistance. From a functionalist perspective, technology non-use focuses on the specific behaviors of individual actors (17); media resistance, from a collective action angle, involves strong life-political overtones, as media resisters address social concerns, fearing that media and communication technologies might undermine moral values, erode democracy through opinion control, damage social cohesion, and harm physical and mental health (18); and digital disconnection emphasizes the contextual and personal needs associated with using ICTs (17).

#### 2.2.2 Digital detox

Digital detox refers to periodic disconnection from social media or instant messaging apps or strategies to reduce digital media engagement (19). This rhetorical term presupposes that ICTs inevitably negatively impact users, leading to a premise of “toxicity,” whereas digital disconnection is neutral when the interaction between humans and technology is explored (11, 20).

#### 2.2.3 Digital minimalism

Digital minimalism refers to concentrating online time on a few carefully selected and optimized activities that strongly support what one values while happily blocking out everything else (21). Digital minimalism is the guiding philosophy for implementing digital disconnection (17).

To address the gap in employee digital disconnection, this study adopted the questionnaire survey method to construct an employee digital disconnection scale with cultural sensitivity, especially for the workplace environment in China. First, through a literature review, four core dimensions were extracted based on the theoretical framework of Verlinden et al. (10): DD POW, DD WOW, DD PDW, and DD WDW. In the subsequent quantitative research stage, the validity of the scale was verified through various quantitative analysis methods, including exploratory factor analysis, confirmatory factor analysis, reliability, convergent validity, discriminant validity, criterion-related validity, and measurement equivalence test. The following text will elaborate on each research link in detail and deeply explore its academic contributions.

## 3 Materials and methods

### 3.1 Participants

This study employed a questionnaire survey method and collected data online from employees in Guangdong Province, Jiangsu Province, Shandong Province, Zhejiang Province, and other provinces. The sample was diverse, covering not only the public sector but also industries such as manufacturing, real estate, construction, software, and commercial services, ensuring industry diversity and representativeness. To improve response rates and data quality, participants were compensated for completing the questionnaire. The survey was distributed twice, with 500 questionnaires each time. After excluding invalid responses (e.g., short completion time, patterned answers), sample 1 included 400 valid questionnaires (80% valid response rate), and sample 2 included 395 valid questionnaires (79% valid response rate). Sample 1 was used for item analysis and exploratory factor analysis, sample 2 was used for confirmatory factor analysis, and the entire dataset was used for reliability analysis. The demographic characteristics are shown in Table 1.

**Table 1:** Demographic characteristics.

Demographics	Classification	Sample 1 (*n* = 400, %)	Sample 2 (*n* = 395, %)
Demographics	Classification	%	%
Gender	Male	39	39.49
	Female	61	60.51
Age	Aged 21 to 30	38	34.43
	Aged 31 to 40	51.25	54.94
	Aged 41 to 50	7	7.59
	51 years old or above	3.75	3.04
Education level	Junior High School and Below	0.75	0.25
	Vocational School and High School	2	2.03
	College degree	7.25	7.59
	Bachelor degree	63.75	60.76
	Graduate degree	26.25	29.37
Years of work	under1 year	1	1.26
	1 to 5 years	26.75	23.29
	5 to 10 years	44.75	45.32
	10 to 15 years	17	18.48
	15 years or above	10.5	11.65

### 3.2 Measures

#### 3.2.1 Employee digital disconnection scale

This scale was developed by Verlinden et al. (10), consisting of 30 items. It covers three dimensions: DD WOW, DD PDW, and DD WDW. A 5-point Likert scale was used (1 = never or not applicable at all, 5 = always or completely applicable). Existing studies have determined family-related scales based on work-related scales. For example, previous research determined the family identity salience scale based on the work identity salience scale (22). In addition, in the original EDDS, the items of DD WOW are basically the same as those of DD WDW, except that the context has changed. Therefore, based on the dimension of DD PDW of EDDS, we developed and designed the dimension of DD POW. The addition of this dimension holds particular significance for understanding employee digital disconnection behaviors within the Chinese cultural context. In the cultural tradition of Chinese society, which emphasizes interpersonal relationships and family ethics, employees’ “digital disconnection from private ICTs outside work” may lead to disengagement from core social networks, triggering anxiety and depressive symptoms (23). Crucially, DD POW becomes a salient occupational construct precisely because the boundary between work and personal life is notably blurred and permeable (24). This means that in practice, employees often find it difficult to clearly divide their disconnected work. The cognitive burden of constantly managing and filtering between work and personal notifications often makes a complete “digital shutdown”—encompassing both work and private ICTs—the most feasible recovery strategy. Consequently, the choice to disconnect from private channels is seldom a purely personal media decision; it is fundamentally shaped by work-induced pressures and recovery needs, solidifying DD POW as a meaningful variable in occupational health research.

Our study followed the “translation-back translation” procedure to first translate all the scales into Chinese and then conduct back translation and verification. A bilingual (Chinese and English) professor of organizational behavior was involved. Two workers who had already entered the workplace were invited to answer the processed measurement questionnaire, providing suggestions on the questionnaire item settings. On the basis of this feedback, further adjustments were made to the items, ultimately resulting in the first draft of the Chinese Version of the Employee Digital Disconnection Scale (Chinese-EDDS), comprising 40 items across four dimensions: DD POW, DD WOW, DD PDW, and DD WDW. A 5-point Likert scale was used (1 = never or not applicable at all, 5 = always or completely applicable).

#### 3.2.2 Job autonomy

Job autonomy refers to the degree of freedom employees have in making decisions during their work and their ability to control their own working methods (25). Given the close relationship between job autonomy and digital disconnection (26), in this study, the Job Autonomy Scale was selected as the validity criterion. Developed by Kirmeyer and Shirom (27), it consists of 7 items and is unidimensional. As presented in “Appendix A,” all items adopted a 5-point Likert scale (1 = strongly disagree, 5 = strongly agree). In this study, the Cronbach’s *α* was 0.837.

### 3.3 Statistical methods

In this study, SPSS 27.0 was used for item analysis, exploratory factor analysis, reliability analysis, and criterion-related validity analysis, and Mplus 8.3 was used for confirmatory factor analysis. Controlling for the effects of an unmeasured latent methods factor (ULMC) indicated that there were no severe common method biases in this study (∆RMSEA = 0.002, ∆SRMR = 0.008, ∆CFI = 0.011, and ∆TLI = 0.005).

## 4 Results

### 4.1 Item analysis

Conduct item analysis on the data of sample 1. Item analysis was performed via item-total correlation and the extreme group method. First, the correlation coefficient between each item score and the total score was used as an indicator for item analysis. The results revealed that the correlation coefficients between all 40 items and the total score were significant (*p* < 0.01). Second, the total questionnaire scores were ranked, and the highest and lowest 27% were divided into high and low groups. An independent-samples *t*-test was used to compare the differences between each item in the high and low groups. The results indicated that the *t*-values ranged from −21.733 to −2.408, and all the values reached significance levels (*p* < 0.05), as shown in Table 2.

**Table 2:** *T*-test for the differences in the high and low groups for each item and their correlation with the total score.

Item	*t*	*r*	Item	*t*	*r*
DD POW1	−2.842**	0.306**	DD PDW1	−17.645***	0.627**
DD POW2	−5.001***	0.412**	DD PDW2	−20.327***	0.674**
DD POW3	−4.566***	0.388**	DD PDW3	−19.092***	0.634**
DD POW4	−6.016***	0.465**	DD PDW4	−20.504***	0.693**
DD POW5	−6.297***	0.500**	DD PDW5	−19.273***	0.676**
DD POW6	−3.668***	0.359**	DD PDW6	−16.971***	0.613**
DD POW7	−5.565***	0.430**	DD PDW7	−21.733***	0.676**
DD POW8	−6.331***	0.493**	DD PDW8	−18.358***	0.653**
DD POW9	−8.088***	0.417**	DD PDW9	−7.962***	0.406**
DD POW10	−4.632***	0.296**	DD PDW10	−6.508***	0.353**
DD WOW1	−16.248***	0.595**	DD WDW1	−2.408*	0.253**
DD WOW2	−17.607***	0.632**	DD WDW2	−4.601***	0.439**
DD WOW3	−16.180***	0.606**	DD WDW3	−5.400***	0.397**
DD WOW4	−17.846***	0.663**	DD WDW4	−4.041***	0.385**
DD WOW5	−16.589***	0.611**	DD WDW5	−4.708***	0.411**
DD WOW6	−15.791***	0.621**	DD WDW6	−3.855***	0.334**
DD WOW7	−18.068***	0.652**	DD WDW7	−4.439***	0.348**
DD WOW8	−19.202***	0.665**	DD WDW8	−6.925***	0.402**
DD WOW9	−9.823***	0.491**	DD WDW9	−4.143***	0.251**
DD WOW10	−5.949***	0.352**	DD WDW10	−3.682***	0.260**

*n* = 400. *** *p* < 0.001, ** *p* < 0.01, * *p* < 0.05. DD POW, digital disconnection from private ICTs outside work; DD WOW, digital disconnection from work-related ICTs outside work; DD PDW, digital disconnection from private ICTs during work; DD WDW, digital disconnection from work-related ICTs during work.

### 4.2 Exploratory factor analysis

Exploratory factor analysis (EFA) was conducted on sample 1 data (*n* = 400). On the basis of the results of the item analysis, EFA was performed on the 40 items retained in sample 1. The relevant indicators (KMO = 0.901, χ^2^ = 10850.779, df = 4,780, and *p* < 0.001) indicate that the data are suitable for EFA.

Principal component analysis and the varimax method were used for analysis. The results revealed that Factor 5 was composed of items 9 and 10 from four dimensions. Because items 9 and 10 in the four contexts have similar wording (both involving “notify” or “I think it’s important”), they naturally aggregated into one factor in the EFA. According to the division principle of the four contexts in the original EDDS (work/nonwork time and work-related/private content) (10), this new factor does not belong to any of the four contexts. Therefore, items 9 and 10 from all four dimensions were removed, resulting in 8 deleted items. EFA was conducted again, following deletion criteria requiring factor loadings above 0.4, at least 3 items per factor, and cross-loading differences not less than 0.20. Items DD WDW8 and DD POW5 were sequentially removed, leaving 30 items. Four common factors with eigenvalues of 9.637, 5.021, 2.739, and 2.300 were extracted, and the cumulative variance contribution rate was 65.658%. The remaining items had loadings between 0.633 and 0.857. After EFA, the Chinese-EDDS has 30 items across four dimensions: factors 1 to 4 correspond to DD PDW, DD WOW, DD POW, and DD WDW. The specific data are presented in Table 3.

**Table 3:** Exploratory factor analysis of digital disconnection.

Item	Factor loading				Communality
	Factor 1	Factor 2	Factor 3	Factor 4	
DD PDW3	0.835				0.751
DD PDW2	0.833				0.765
DD PDW7	0.820				0.742
DD PDW1	0.816				0.717
DD PDW6	0.812				0.701
DD PDW8	0.794				0.680
DD PDW4	0.778				0.703
DD PDW5	0.763				0.702
DD WOW3		0.857			0.773
DD WOW1		0.846			0.747
DD WOW6		0.837			0.747
DD WOW2		0.822			0.741
DD WOW5		0.810			0.721
DD WOW4		0.802			0.714
DD WOW7		0.760			0.686
DD WOW8		0.668			0.578
DD POW2			0.803		0.676
DD POW3			0.796		0.667
DD POW1			0.771		0.622
DD POW7			0.717		0.584
DD POW8			0.707		0.566
DD POW6			0.699		0.528
DD POW4			0.690		0.526
DD WDW6				0.770	0.635
DD WDW2				0.768	0.657
DD WDW3				0.754	0.603
DD WDW4				0.739	0.595
DD WDW1				0.721	0.587
DD WDW5				0.711	0.556
DD WDW7				0.633	0.427
Eigenvalue	9.637	5.021	2.739	2.300	
variance contribution rate %	32.123	16.737	9.130	7.668	
Cumulative variance contribution rate %	32.123	48.860	57.990	65.658	

*n* = 400. DD POW, digital disconnection from private ICTs outside work; DD WOW, digital disconnection from work-related ICTs outside work; DD PDW, digital disconnection from private ICTs during work; DD WDW, digital disconnection from work-related ICTs during work.

### 4.3 Confirmatory factor analysis

Using data from sample 2 (*n* = 395), a confirmatory factor analysis (CFA) was conducted. The CFA fitting result of the 4-factor model is better than that of the 8-factor model (see Table 4). This indicates that the 4-factor model is more suitable for China. This might be caused by cultural differences and the degree of sample diversity. The research of EDDS mainly used samples from the European public sector (10). The samples we used were not limited by industry and were more diverse.

**Table 4:** CFA results and alternative models.

Model	χ^2^	df	χ^2^/df	CFI	TLI	RMSEA	SRMR
One-factor model	4925.784	405	12.16	0.466	0.426	0.168	0.177
Four-factor model	1169.625	399	2.931	0.909	0.901	0.070	0.056
Eight-factor model	2818.361	712	3.958	0.802	0.784	0.087	0.099
Second-order factor	1228.113	401	3.063	0.902	0.894	0.072	0.082

*n* = 395. One-factor model: DD POW + DDWOW + DD PDW + DD WDW. Four-factor model: DD POW, DDWOW, DD PDW, DD WDW. Eight-factor model: split each context-level component into two sub-dimensions (technological and communication). Second-order factor: four first-order factors loading on one “Digital Disconnection” factor. DD POW, digital disconnection from private ICTs outside work; DD WOW, digital disconnection from work-related ICTs outside work; DD PDW, digital disconnection from private ICTs during work; DD WDW, digital disconnection from work-related ICTs during work.

### 4.4 Reliability analysis

Reliability analysis revealed that the Cronbach’s *α* for the total Chinese-EDDS was 0.925, with the Cronbach’s α for each dimension ranging between 0.875 and 0.944. The Cronbach’s α of the DD POW factor, the DD WOW factor, the DD PDW factor, and the DD WDW factor were 0.888, 0.938, 0.944, and 0.875, respectively, as shown in Table 5.

**Table 5:** Reliability analysis results.

Metric	Chinese-EDDS	DD POW	DD WOW	DD PDW	DD WDW
Cronbach’s α	0.925	0.888	0.938	0.944	0.875

*n* = 795. DD POW, digital disconnection from private ICTs outside work; DD WOW, digital disconnection from work-related ICTs outside work; DD PDW, digital disconnection from private ICTs during work; DD WDW, digital disconnection from work-related ICTs during work.

### 4.5 Convergent validity and discriminant validity analysis

Using sample 2 (*n* = 395) for convergent validity and discriminant validity analysis, as shown in Table 6, the composite reliability (CR) of the four-factor model was greater than 0.7, and the average variance extracted (AVE) was greater than 0.5, indicating that the Chinese-EDDS has good convergent validity. The square root of the AVE for each factor was greater than the correlation coefficient values between each pair of factors, demonstrating good discriminant validity of the scale.

**Table 6:** Convergent validity analysis.

Metric	DD POW	DD WOW	DD PDW	DD WDW
CR	0.894	0.940	0.947	0.880
AVE	0.546	0.663	0.691	0.515

*n* = 395. DD POW, digital disconnection from private ICTs outside work; DD WOW, digital disconnection from work-related ICTs outside work; DD PDW, digital disconnection from private ICTs during work; DD WDW, digital disconnection from work-related ICTs during work.

### 4.6 Criterion-related validity analysis

Job autonomy was used as the criterion tool. As shown in Table 7, the total score of the Chinese-EDDS was significantly positively correlated with the criterion tool, and the scores of each dimension of the Chinese-EDDS were also significantly positively correlated with the criterion tool. All correlation coefficients ranged between 0.105 and 0.318, which aligns with the expected hypothesis, indicating that the Chinese-EDDS has good criterion-related validity.

**Table 7:** Criterion-related validity analysis.

Variable	DD POW	DD WOW	DD PDW	DD WDW	Chinese-EDDS
Job Autonomy	0.105*	0.140**	0.318***	0.136*	0.246**

*** *p* < 0.001, ** *p* < 0.01, * *p* < 0.05, DD POW, digital disconnection from private ICTs outside work; DD WOW, digital disconnection from work-related ICTs outside work; DD PDW, digital disconnection from private ICTs during work; DD WDW, digital disconnection from work-related ICTs during work.

### 4.7 Measurement equivalence test

Sample 2 (*n* = 395) was used to conduct cross-group measurement equivalence testing for gender (male = 0, female = 1), and the model fit indices for each model are shown in Table 8. The results indicate that the configural invariance model, weak invariance model, and strong invariance model of the Chinese-EDDS all have good fit indices (∆CFI ≤ 0.01, ∆TFI ≤ 0.01, and ∆RMSEA≤0.01). This scale exhibits cross-group measurement invariance.

**Table 8:** Chinese-EDDS cross-gender measurement equivalence analysis.

Model	χ^2^(df)	CFI	TLI	RMSEA	∆CFI	∆TLI	∆RMSEA
Configural invariance	1556.561 (794)	0.910	0.901	0.070			
Weak invariance	1584.749 (820)	0.910	0.904	0.069	0.000	−0.003	0.001
Strong invariance	1603.684 (846)	0.911	0.908	0.067	−0.001	−0.004	0.002

## 5 Discussion

The main conclusions of this study are as follows. First, the scale framework of this study is based on two core dimensions: the content of disconnected ICTs (work-related and private) and the scenarios of disconnection behaviors (during work and outside work). Through cross-combination, four dimensions of the employee digital disconnection scale in the Chinese context were determined: digital disconnection from private ICTs outside work (DD POW, 7 items), digital disconnection from work-related ICTs outside work (DD WOW, 8 items), digital disconnection from private ICTs during work (DD PDW, 8 items), and digital disconnection from work-related ICTs during work (DD WDW, 7 items). There are a total of 30 measurement items, as shown in “Appendix A.” The scale has good reliability and validity. This scale aims to measure employees’ intentional digital disconnection behaviors rather than the degree of digital disconnection.

Second, this study supplements the fourth dimension of the employee digital disconnection scale. Verlinden et al. (10) categorized employee digital disconnection into four contexts. They argued that digital disconnection from private ICTs outside work (DD POW) is not unique to employees but applies to any media user (10). Therefore, they developed an EDDS for only three of the other contexts and did not create one for this specific scenario. However, this reasoning implies that employees encounter this disconnection context. To measure employee digital disconnection behavior more comprehensively, this study supplements the scale for DD POW.

Finally, digital disconnection from work-related ICTs outside work (DD WOW) particularly highlights the uniqueness of the Chinese cultural context. The items under this dimension, such as “I completely turn off the devices I use for work,” directly challenge the prevalent always-on culture in Chinese workplaces. The collectivist culture in China emphasizes individual interests being subordinate to collective interests, leading people to experience stronger social expectations (15). Therefore, achieving DD WOW is challenging; as such, proactive boundary-setting behaviors require overcoming greater societal pressure. In contrast, digital disconnection from private ICTs during work (DD PDW) aligns with Chinese workplace norms of dedication and self-discipline. Items under this dimension, such as “I completely shut down the devices I use for personal matters,” conform to organizational expectations of employee focus. This is because employees who engage in work-unrelated activities during work hours consume available resources and weaken their ability to subsequently engage in proactive behaviors (28). These two dimensions collectively reveal how China’s collectivist culture profoundly shapes employees’ digital boundary management strategies between work and private domains.

Unlike the “context-strategy” two-tier structure of the original Western scale, in this study, a more concise four-factor structure was discovered in the sample of Chinese employees, that is, employees directly organized their disconnection behaviors according to four scenarios. We believe that there are real cultural reasons behind this structural difference. Chinese culture is a typical high-context culture. In high-context cultures, language is relatively tactful and emphasizes conveying information through non-verbal cues (29). In this mode, when employees are thinking about how to disconnect, the first thing they consider is “in what situation am I?” rather than choosing between “using technical methods” or “using communication methods.” In actual response, they often naturally combine turning off notifications (technical) with greeting colleagues (communication) as an overall approach to handling the same matter. Therefore, in terms of data, these behaviors are more stably attributed to the same “context” factor rather than being separated into two separate dimensions.

### 5.1 Theoretical implications

First, this study further clarifies the distinctions and connections between digital disconnection and related concepts. By sorting out the differences and connections between digital disconnection and technology non-use, media resistance, digital detox, and digital minimalism, its theoretical boundaries are further clarified. Unlike concepts such as “digital detox,” which presupposes that ICTs inevitably have negative effects (20), digital disconnection assumes that ICTs are neutral, with a focus on the dynamic interaction between humans and technology. This characteristic is reflected in the measurement items across the four dimensions. Whether it involves technical restrictions for private or work communications during work or outside work (such as turning off notifications or enabling airplane mode) or proactive communication behaviors (such as informing others of unavailability) reflects employees’ autonomous adjustment strategies in different contexts without entirely negating technology. This perspective breaks through the premise that ICTs inevitably have negative effects on users, revealing the dual-path mechanism (11) through which digital disconnection may simultaneously trigger both positive and negative impacts, thereby providing a clear framework for a deeper understanding of the theoretical uniqueness and complexity of this construct.

Second, in this study, a four-dimensional theoretical structure of employee digital disconnection in the Chinese context is constructed and validated. This structure not only identifies the cross-context of work/nonwork time and work-related/private content but also reveals that digital disconnection is not a vague one-dimensional concept but a context-driven, multilayered construct (10). This provides a theoretical framework and measurement foundation for future research, enabling in-depth exploration of the differentiated antecedents and consequences of digital disconnection in various contexts, thereby promoting the transition from qualitative to empirical research in this field.

Third, this study provides a comprehensive perspective on digital disconnection in the Chinese context, presenting a critical addition to existing research focused mainly on the Western context. Nassen et al. (11) clarified the concept of voluntary digital disconnection, but existing studies are predominantly based on Western contexts (30); thus, the related discussions in the Chinese context are insufficient. Given China’s collectivist culture, this study, which is grounded in the local context, analyzes four scenarios of employee digital disconnection, urging domestic academics to focus on and explore this topic further. Additionally, on the basis of Verlinden et al.’s (10) EDDS, a Chinese-EDDS was developed and validated in the Chinese context in response to calls for localized tool development (7, 17) and the use of diverse samples, providing reliable measurement foundations for empirical research in China.

Finally, digital well-being is a concept that emphasizes the importance of mental, physical, and emotional health in individuals’ interactions with technology (31). It seeks to mitigate the adverse effects of technology while fostering a more balanced and effective utilization of technological tools in social contexts (32). Recent research indicates that digital disconnection is not an isolated act but a crucial part of a series of strategies for achieving digital well-being (33). Therefore, the revised scale in this study goes beyond merely measuring the “digital disconnection” behavior itself, and has become a key measurement tool that connects the micro-individual behavior with the macro-health construct.

### 5.2 Management implications

First, organizational managers should pay attention to employees’ digital disconnection behaviors. When employees actively engage in digital disconnection, managers need to focus on granting them greater job autonomy, allowing them to flexibly and independently allocate work time and attention according to their own rhythms and needs, thereby achieving a healthier and more sustainable work environment.

Second, organizational managers should emphasize the technological application and development of employees’ digital disconnection. By providing tools with multiple self-configurable disconnection modes (such as the work mode, rest mode, or deep focus mode), employees can autonomously choose the intensity and frequency of digital connectivity in various scenarios while limiting the scope and quantity of available functions. This promotes the evolution of the employee-technology-society-information ecosystem in a “people-oriented” sustainable development direction.

Finally, organizations should foster a cultural atmosphere that supports employees’ digital disconnection. Given China’s collectivist cultural background, employees often experience disconnection anxiety because of fears of detaching from the collective. Therefore, organizations should actively develop a culture that understands and respects individual digital boundaries. Managers can publicly affirm the value of reasonable digital disconnection and clearly delineate the boundaries between work hours and private time. For example, managers advocate that employees avoid sending work messages through tools such as WeChat Work or DingTalk outside work or explicitly inform employees that they do not need to reply to work messages during their private time. This cultural shaping not only helps alleviate employees’ anxiety and guilt associated with digital disconnection but also enhances their sense of identification with and belonging to the organization.

### 5.3 Limitations and future research

The study is not without limitations. First, the data come from employee self-assessments, so there is a potential social desirability bias, posing a risk of common method bias. Future research could incorporate measurement methods using objective data (such as the usage time of different software, the number and types of notifications closed or silenced, etc.) to further enhance the reliability and comprehensiveness of the study findings. Additionally, we relied on cross-sectional data, but to clarify the long-term consequences of digital disconnection, longitudinal research is needed.

Second, this study was conducted solely within the Chinese context. Influenced by the national cultural background and the institutional environment, the manifestation dimensions of employees’ digital disconnection and their psychological mechanisms may have regional specificity, and the cross-cultural generalizability of the research conclusions remains to be tested. Future studies could select countries or regions with distinct cultural characteristics for cross-cultural comparisons, further validating the applicability and robustness of the scales developed in this study. This would expand the external validity of digital disconnection theory and provide an empirical foundation for constructing a more universally explanatory theoretical framework.

Third, approximately 61% of the two samples in this study were female. This imbalance in proportion may be caused by multiple factors. The main reason might stem from the systematic recruitment bias of the online survey panel. While this distribution incidentally mirrors that of the original validation study (10), enhancing comparability, it may limit the generalizability of our findings to male employee populations. Nonetheless, we proactively addressed this concern by conducting a measurement equivalence analysis across genders. The results confirmed configural, weak, and strong invariance, indicating that the factor structure and the meaning of the latent constructs are equivalent between males and females. This provides strong evidence that the observed relationships among variables are not an artifact of the uneven sample split, thereby substantially mitigating validity concerns related to the gender imbalance.

Finally, because digital disconnection is a newly introduced construct, its theoretical system is still immature. The causes of digital disconnection are complex and diverse, and scholars are divided on the results of digital disconnection (11). Therefore, efforts to clarify the antecedent (e.g., personal characteristics, job characteristics, and organizational factors) and outcome variables (e.g., employee mental health and individual thriving) of digital disconnection and reveal its underlying mechanisms will contribute to the development of the digital disconnection field.

## Data Availability

The raw data supporting the conclusions of this article will be made available by the authors, without undue reservation.
