# Supplementary material for: Chinese version of the employee digital disconnection scale: development and validation
Source: Front Public Health. 2026 Feb 3;14:1762986. doi: 10.3389/fpubh.2026.1762986 (PMC12909549; doi:10.3389/fpubh.2026.1762986)
Supplement: Supplementary file 1 [file Data_Sheet_1.docx]

**Appendix A**

**Job autonomy**

1. I had freedom to decide what to do.
2. I had freedom to decide how to do my own job.
3. I had responsibility for deciding how the job got done.
4. I had a lot to say about what happened on the job.
5. I had latitude to decide when to take breaks.
6. I had latitude to decide with whom I worked.
7. I had latitude to decide the speed at which I worked.

**Chinese Version of the Employee Digital Disconnection Scale**

1. **Digital disconnection from private ICTs outside work (DD POW)**

How often do you do the following outside your standard work hours? (Never - rarely - sometimes - often – always)

1. I don’t react to private notifications (e.g., pop-ups or sound alerts of messages, emails, or calls from family members, friends...).
2. I disable private notifications (e.g., pop-ups or sound signals of messages or calls from family members, friends...).
3. I keep private programs or applications closed, or I don’t open them (e.g., chats, social media, news apps...).
4. I turn off the connection on the devices I use for personal matters (e.g., airplane mode, turning off Wi-Fi or mobile data...).
5. I keep my devices for private use out of sight or don’t bring them to certain activities (e.g., smartphone during a meeting).

To what extent do the following statements apply to you? (Not at all - to a limited extent - to an average extent - to a great extent - completely)

1. I agree with people in my personal environment that I'm not reachable via smartphone during my work hours.
2. I indicate in my communication channels that I don't react to messages or calls from people in my personal environment during my work hours (e.g., through an absence message or offline status).
3. **Digital disconnection from work-related ICTs outside work (DD WOW)**

How often do you do the following outside your standard work hours? (Never - rarely - sometimes - often – always)

1. I ignore work-related notifications (e.g., pop-ups or sound alerts of messages, emails, or calls from colleagues, my boss, clients...).
2. I switch off work-related notifications (e.g., pop-ups or sound signals of messages or calls from colleagues, my boss, clients...).
3. I keep work-related programs or applications closed, or I don’t open them (e.g., chats, emails, intranet...).
4. I put the devices I use for work (e.g., laptop, cell phone, iPad...) in an offline mode (e.g., airplane mode, disabling Wi-Fi or mobile data, via an app or focus mode...).
5. I completely turn off the devices I use for work (e.g., laptop, cell phone, iPad...).
6. I put away the devices I use for work (e.g., laptop, cell phone, iPad...) or don’t take them with me to certain activities.

To what extent do the following statements apply to you? (Not at all - to a limited extent - to an average extent - to a great extent - completely)

1. I agree with people within my professional network that I am not accessible through my computer or smartphone off hours.
2. By a message in my work communication channels, I inform colleagues, my boss, and/or clients that I am unavailable outside working hours (e.g., through an absence message or offline status).
3. **Digital disconnection from private ICTs during work (DD PDW)**

How often do you do the following during your standard work hours? (Never - rarely - sometimes - often – always)

1. I don’t react to private notifications (e.g., pop-ups or sound alerts of messages, emails, or calls from family members, friends...).
2. I disable private notifications (e.g., pop-ups or sound signals of messages or calls from family members, friends...).
3. I keep private programs or applications closed, or I don’t open them (e.g., chats, social media, news apps...).
4. I turn off the connection on the devices I use for personal matters (e.g., airplane mode, turning off Wi-Fi or mobile data...).
5. I completely shut down the devices I use for personal matters (e.g., smartphone...).
6. I keep my devices for private use out of sight or don’t bring them to certain activities (e.g., smartphone during a meeting).

To what extent do the following statements apply to you? (Not at all - to a limited extent - to an average extent - to a great extent - completely)

1. I agree with people in my personal environment that I'm not reachable via smartphone during my work hours.
2. I indicate in my communication channels that I don't react to messages or calls from people in my personal environment during my work hours (e.g., through an absence message or offline status).
3. **Digital disconnection from work-related ICTs during work (DD WDW)**

How often do you do the following during your standard work hours? (Never - rarely - sometimes - often – always)

1. I don’t immediately react to work-related notifications (e.g., pop-ups or sound alerts of messages, e-mails, or calls from colleagues, my boss, customers...).
2. I disable work-related notifications (e.g., pop-ups or sound signals of messages or calls from colleagues, my boss, clients...).
3. I keep certain work-related programs or applications closed, or don’t open them (e.g., chats, emails, intranet...).
4. I put certain devices I use for work in an offline mode (e.g., airplane mode, disabling Wi-Fi or mobile data ...).
5. I entirely close certain devices I use for work (e.g., laptop, cell phone, iPad ...).
6. I set aside certain devices I use for work or deliberately don’t bring them to certain activities (e.g., smartphone or laptop during a meeting).

To what extent do the following statements apply to you? (Not at all - to a limited extent - to an average extent - to a great extent - completely)

1. I agree with people in my professional network on the moments during the work day they can’t reach me.
